# Supplementary material for: Development of Energy-Efficient Superhydrophobic Polypropylene Fabric by Oxygen Plasma Etching and Thermal Aging
Source: Polymers (Basel). 2020 Nov 23;12(11):2756. doi: 10.3390/polym12112756 (PMC7700148; doi:10.3390/polym12112756)
Supplement: Supplementary file 1 [file polymers-12-02756-s001.pdf]

Supporting Information

Development of Energy-efficient Superhydrophobic Polypropylene Fabric by Oxygen Plasma Etching and Thermal Aging

Shinyoung Kim[1], Ji-Hyun Oh[1,2] and Chung Hee Park[1]*
[1]Department of Textiles, Merchandising and Fashion Design, Seoul National University, 08826 Republic of Korea
[2]Department of Chemical and Biomolecular Engineering, North Carolina State University, Raleigh, North Carolina, 27695, United States

SEM image	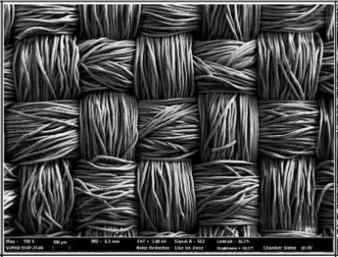	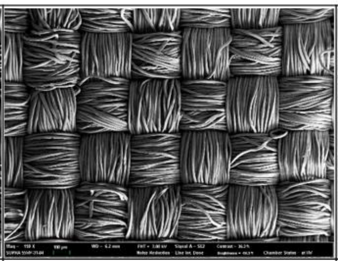	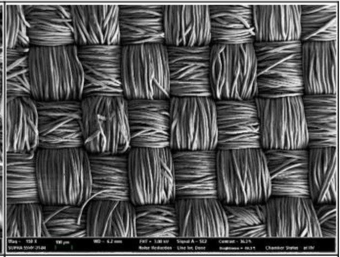
Weave density (in inch)	62/inch × 62/inch	72/inch × 72/inch	82/inch × 80/inch

Figure S1. Characteristics of specimens according to weave density.

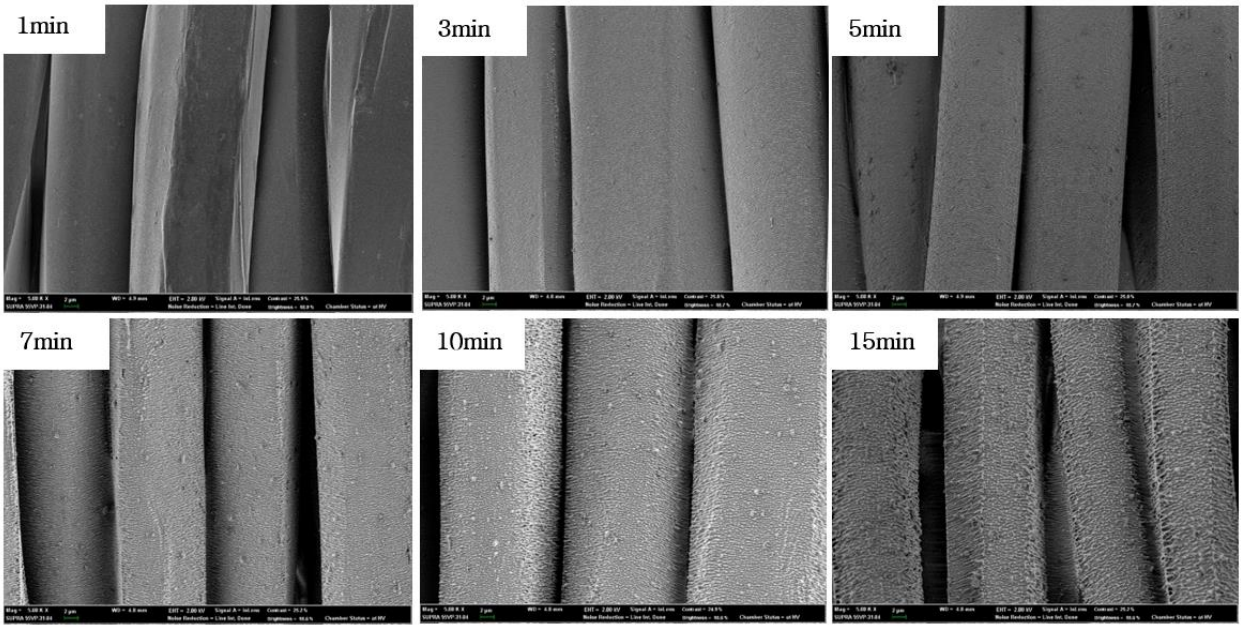

Figure S2. SEM images of the plasma-etched specimens for 1,3,5,7,10, 15 mins. (×5,000, top view).

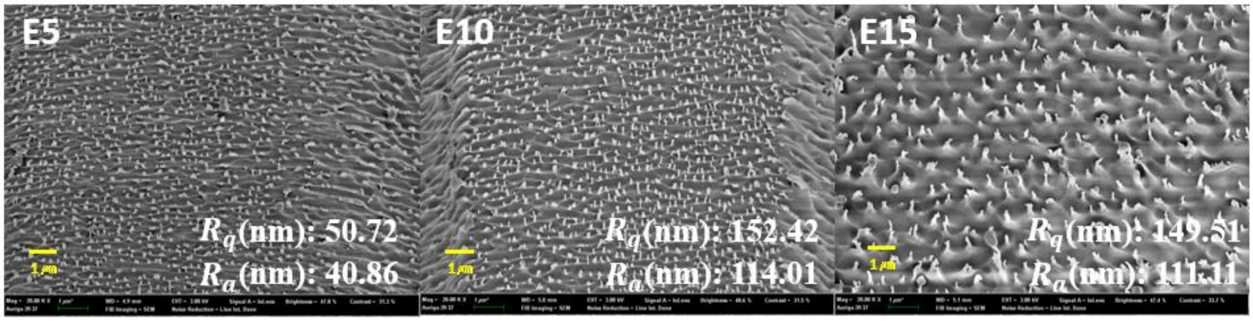

Figure S3. SEM images and nano roughness(AFM) of the plasma-etched for 5,10,15 mins. And thermal aging for 24h.

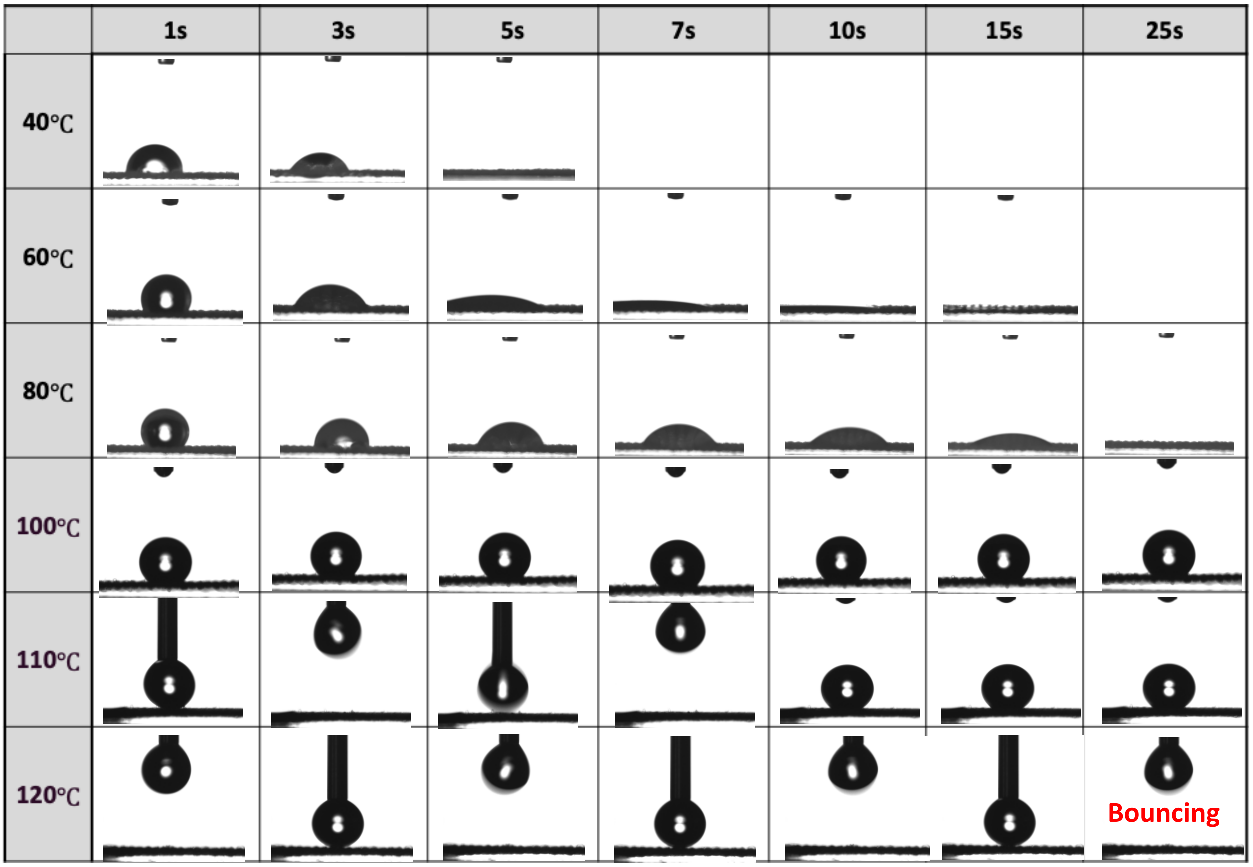

Figure S4. Photographs of water droplets on the specimens treated with plasma etching for 10min and thermal aging for 24h depending on various temperatures and time lapse.

Table S1. Glass transition temperature and melting temperature of PP fabric and PP film.

	PP fabric	PP film
Tg(°C)	1.9	163.0
Tm(°C)	1.4	157.8

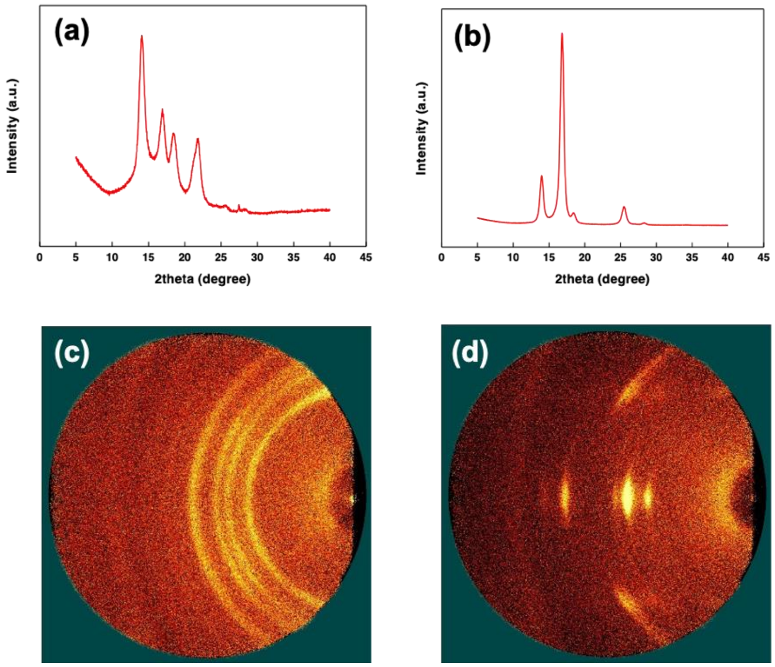

Figure S5. Degree of crystallinity of PP film (**a**) and PP fabric (**b**) and degree of orientation form XRD for PP film (**c**) and PP fabric (**d**).

Table S2. Water contact angle, shedding angle and sliding angle of untreated PP fabric and PP film specimens

	**fabric**			**film**
	**62/inch × 62/inch**	**72/inch × 72/inch**	**82/inch × 80/inch**	
**WCA**	143.5 ± 1.3	138.7±1.9	133.3 ± 1.7	103.8 ± 1.5
**ShA**	17.2±0.7	21.0 ± 0.6	32.8 ± 0.7	>90.0
**SA**	31.6 ± 1.0	43.4 ± 2.2	57.8 ± 3.2	>90.0

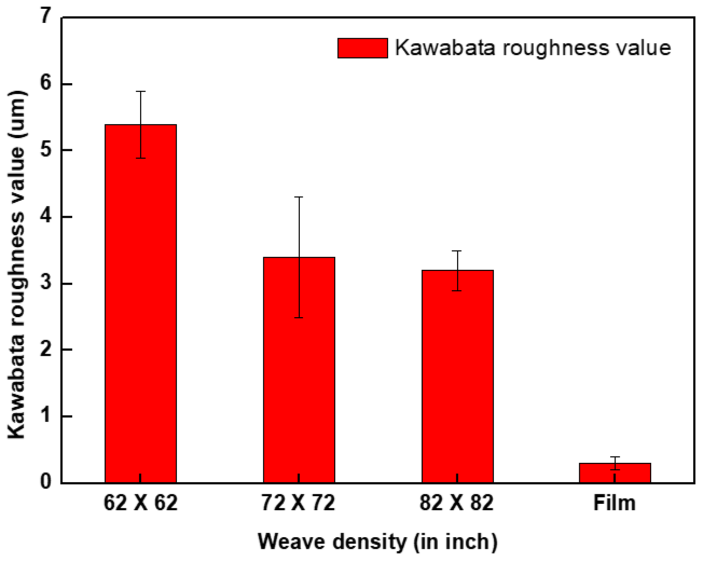

Figure S6. Kawabata surface roughness value of non-plasma etched specimen.

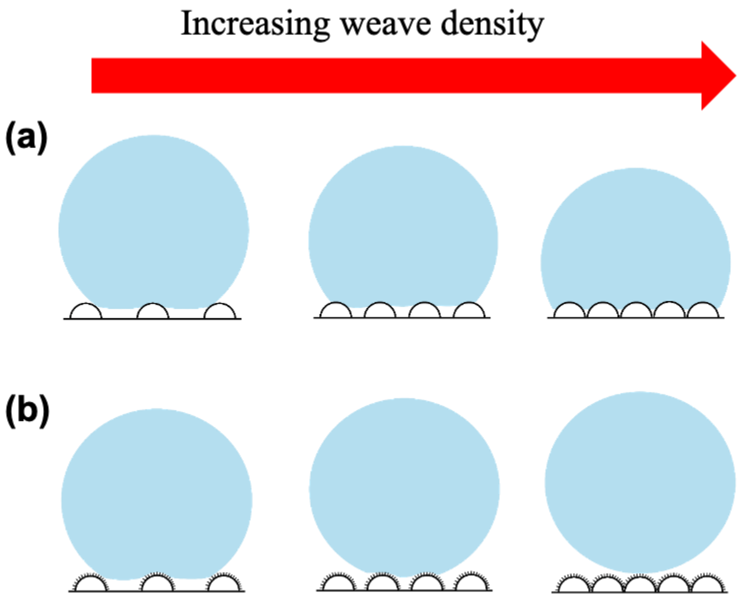

Figure S7. A schematic of water droplets on the untreated PP fabric (**a**) and plasma-etched PP fabric for 15 min (**b**) having different weave densities. (left: 62/inch×62/inch, center: 72/inch×72/inch and right: 82/inch×80/inch).
